# Variations in Health-Related Quality of Life (HRQoL) and survival 1 year after stroke: five European population-based registers

**DOI:** 10.1136/bmjopen-2014-007101

**Published:** 2015-06-01

**Authors:** Salma Ayis, Ian Wellwood, Anthony G Rudd, Christopher McKevitt, David Parkin, Charles D A Wolfe

**Affiliations:** 1 Division of Health and Social Care Research, King's College London, London, UK; 2 NIHR Biomedical Research Centre at Guy's & St Thomas’ NHS Foundation Trust and King's College London, London, UK; 3 Department of Public Health and Primary Care, Cambridge Institute of Public Health, University of Cambridge School of Clinical Medicine, Cambridge, UK

## Abstract

**Objective:**

There were two main objectives: to describe and compare clinical outcomes and Patient-Reported Outcome Measures (PROMs) collected using standardised procedures across the European Registers of Stroke (EROS) at 3 and 12 months after stroke; and to examine the relationship between patients’ Health-Related Quality of Life (HRQoL) at 3 months after stroke and survival up to 1 year across the 5 populations.

**Design:**

Analysis of data from population-based stroke registers.

**Setting:**

European populations in Dijon (France); Kaunas (Lithuania); London (UK); Warsaw (Poland) and Sesto Fiorentino (Italy).

**Participants:**

Patients with ischaemic or intracerebral haemorrhage (ICH) stroke, registered between 2004 and 2006.

**Outcome measures:**

(1) HRQoL, assessed by the physical component summary (PCS) and mental component summary (MCS) of the Short-Form Health Survey (SF-12), mapped into the EQ-5D to estimate responses on 5 dimensions (mobility, activity, pain, anxiety and depression, and self-care) and utility scores. (2) Mortality within 3 months and within 1 year of stroke.

**Results:**

Of 1848 patients, 325 were lost to follow-up and 500 died within a year of stroke. Significant differences in mortality, HRQoL and utility scores were found, and remained after adjustments. Kaunas had an increased risk of death; OR 2.34, 95% CI (1.32 to 4.14) at 3 months after stroke in Kaunas, compared with London. Sesto Fiorentino had the highest adjusted PCS: 43.54 (SD=0.96), and Dijon had the lowest adjusted MCS: 38.67 (SD=0.67). There are strong associations between levels of the EQ-5D at 3 months and survival within the year. The trend across levels suggests a dose–response relationship.

**Conclusions:**

The study demonstrated significant variations in survival, HRQoL and utilities across populations that could not be explained by stroke severity and sociodemographic factors. Strong associations between HRQoL at 3 months and survival to 1 year after stroke were identified.

**Strengths and limitations of this study**

- Standardisation of training, measures and methods of data collection across all populations ensures a fair comparison between populations, which was lacking in previous studies.
- The study represents a rich source of information for a range of important outcomes, including clinical and Patient-Reported Outcome Measures (PROMs), in five European countries. To our knowledge, the data have not been updated by more recent comparable studies.
- Limitations include the relatively short follow-up period, limiting the inference on survival, and the associations of perception of health and survival up to 1 year after stroke.
- The variations in outcomes remained unexplained despite the use of standardised methods and measures across populations. This suggests a need for further research that will specially examine factors, other than those we adjusted for, that may influence stroke outcomes. These would assist in designing clinical trials and interpreting large-scale comparisons of observational studies.
- The EQ-5D scores were estimated from the Short-Form Health Survey (SF-12) and not measured directly.

## Introduction

Stroke is a long-term condition with substantial impact on physical and mental health.^1–3^ With ageing populations in most European countries and advances in healthcare, more people are expected to live with stroke long-term sequelae. Measures that assess all aspects of Health-Related Quality of Life (HRQoL), such as Patient-Reported Outcome Measures (PROMs) that reflect the evaluation of health from patients’ own perspectives, are therefore becoming increasingly important for healthcare researchers, providers and policymakers. These data are, however, not routinely collected except in special cases such as the PROMs programme in UK's National Health Service (NHS), and are often collected with inadequate rigour.^4–6^ Limitations in data, small sample sizes, lack of comparison groups, and the use of different measures and methods are among the reasons behind the gap in knowledge on PROMs for long-term conditions.

In cardiovascular disease and stroke, these measures provide information that could be used in clinical decision-making to improve the quality of patients’ care.^5^ ^7^ ^8^ With the increased recognition of patients’ perspectives in the evaluation of healthcare, the focus on clinical outcomes has shifted to include instruments, such as the EQ-5D, developed by the EuroQol group.^9^

The EQ-5D is particularly useful for the evaluation of health over time, for comparisons between populations, the calculation of Quality-Adjusted Life Years (QALYs) and cost-effectiveness of treatments. For example, in the UK, the National Institute for Health and Care Excellence (NICE) advocates the use of EQ-5D to generate QALYs to assess health technology and inform reimbursement, and this is also used in the NHS PROMs programme.^8^ ^10^

This study describes and compares clinical outcomes and HRQoL across the European Registers of Stroke (EROS) populations at 3 and 12 months after stroke. It also examines the associations between HRQoL reported at 3 months and survival 1 year after stroke.

## Methods

EROS was established to study the relationship between costs, resource use and outcomes for patients with stroke across European countries. The settings and methods of case ascertainment in this study have been described previously.^11^ ^12^ Briefly, population-based stroke registers were established in six selected European countries representing populations in central (Dijon, France), southern (Sesto Fiorentino, Italy; Menorca, Spain), eastern (Kaunas, Lithuania; Warsaw, Poland) and western Europe (London, UK), and an overall source population of 1 087 048 inhabitants. Centres were selected on the basis of previous experience in running stroke registers and were, therefore, not necessarily representative of their countries as a whole. The study was approved by ethics committees of each of the centres involved.

In order to provide broad perspectives on health outcomes after stroke, HRQoL data were collected using standardised procedures in addition to clinical data.

### Inclusion and exclusion criteria

All patients with a first-ever stroke were identified using overlapping sources of information. Patients admitted to hospitals were identified by screening all acute hospitals serving the source population, including reviews of acute wards, checks of brain imaging referrals, and reviews of hospital discharge registers, by the study team. Patients who were not admitted to hospital were identified by regular screening of all primary care facilities in the study area. In addition, nursing homes and community therapists in the study were contacted and death certificates were checked regularly. Patients with subarachnoid haemorrhage were excluded from our analyses as the evidence base for management differs significantly from those with other types of stroke.

### Data collection

Data were collected between 2004 and 2006, beginning in May 2004 in Dijon, London and Menorca; in June 2004 in Kaunas and Sesto Fiorentino; and in January 2005 in Warsaw. The study population comprised 1848 first-ever stroke patients with cerebral infarction or intracerebral haemorrhage (ICH), and was followed up for 1 year. Stroke was defined according to the WHO definition^13^ with or without brain imaging confirmation. Stroke was classified as cerebral infarction or ICH based on brain imaging within 30 days of stroke onset. Patients without pathological confirmation of stroke subtype were unclassified. Data collection was standardised across all centres and included patient sociodemographic characteristics (age, sex, employment status (full time, part time, unemployed, unable to work due to disability), prestroke activities of daily living (Barthel Index (BI))^14^ and living conditions prestroke (private household alone, private household with others, supportive environment)). All centres received standardised training on the data collection tools before the study began. Data were also collected on clinical markers of the case mix: the Glasgow Coma Scale (GCS);^15^ and a validated six simple variable (SSV) model that included age, prestroke function and living circumstances, the verbal component of the GCS, arm power and the ability to walk.^16^ ^17^

### Outcome measures

The primary outcome measures were mortality within a year of stroke, and HRQoL at 3 and 12 months after stroke. HRQoL was assessed by the Short-Form Health Survey (SF-12), a standardised instrument with established psychometric validity which measures eight health domains: physical functioning; role limitations due to physical health; bodily pain; general health; vitality (energy/fatigue); social functioning; role limitations due to emotional health; and mental health (psychological distress and psychological wellbeing).^18^ The SF-12 generates two main scales, the physical component summary (PCS) and the mental component summary (MCS), using standard scoring procedures based on regression weights, and the authors of the SF-12 recommend using the PCS and MCS rather than individual domain scores.^19^ PCS and MCS were analysed as continuous variables.

The EQ-5D is a widely used self-report instrument for describing a person's health state, covering five dimensions: mobility, self-care, usual activities, pain, and anxiety and depression, each with 3 levels (no problem, some problem, extreme problem). The health state profile that it generates can be assigned an index score, often called a utility value, which represents preferences for that profile derived from a general population survey. The index has a maximum value of 1 for full health according to the profile; an anchor of 0 for a state equivalent to being dead; and values less than 0 for states regarded as worse than being dead.^20^ The utility values have been generated by asking members of the general public to consider health states described by EQ-5D, which they may or may not have experienced, and to value those states using techniques such as time trade-off (TTO) and visual analogue scales (VAS).^20^ ^21^ There are value sets or ‘tariffs’ available for many countries, which show the value of each possible health state.^22^ A response mapping algorithm based on Monte Carlo simulation, averaging estimates over 1000 samples, was used to predict responses to each of the EQ-5D from SF-12 responses and these were used to calculate utility values.^19^ ^23^ ^24^ The algorithm was derived using multinomial response mapping , validated internally and externally using the 2000 US Medical Expenditure Panel Survey (MEPS), generating index values for eight countries: the UK, the USA, Spain, Germany, the Netherlands, Denmark, Japan and Zimbabwe.^22^ For this study, the UK values were used and Spain's values were used for a sensitivity analysis.

## Statistical analysis

Categorical variables were compared across populations using χ^2^ test, and continuous variables were compared using analysis of variance (ANOVA) or Kruskal-Wallis. Multivariable logistic regression models were used to investigate associations between death after stroke, and a range of predictors and potential confounders. These included sociodemographic factors (gender, employment and living conditions) and previously identified predictors: prestroke BI score, age, the verbal components of GCS, and motor deficit (arm power and the ability to walk).^16^ ^25^ The verbal component of GCS was dichotomised between patients who were verbally oriented, scoring 5 on admission and others who scored less than 5.^26^ BI scores prior to stroke were categorised as 0–19 indicating physical dependency, and 20 for full independence.^27^ A swallowing test and whether the patient has been incontinent or catheterised in the acute phase were used as potential additional predictors. Each model included population in addition to the other predictors that have shown association with the outcome in a univariate analysis with p≤0.20. Deaths within 72 h of stroke were excluded in a sensitivity analysis, as these were likely to have different characteristics and severity, and final models were refitted. Responses to EQ-5D at 3 months were treated as potential predictors of death within 1 year after stroke. The logistic model was chosen for the analysis. The mean PCS and MCS at 3 months and 1 year after stroke were compared across populations using ANOVA. The mixed effect models were also used to compare populations by summarising the repeated measurements of PCS, MCS and utilities at 3 and 12 months after stroke, adjusting for case mix and sociodemographic factors. Mean adjusted estimates for the five populations were predicted and displayed graphically. The software STATA (12.0)^28^ was used for all analyses. Results are based on complete cases only.

## Results

### Variations in survival to 3 months and 1 year after stroke

**Figure 1:**
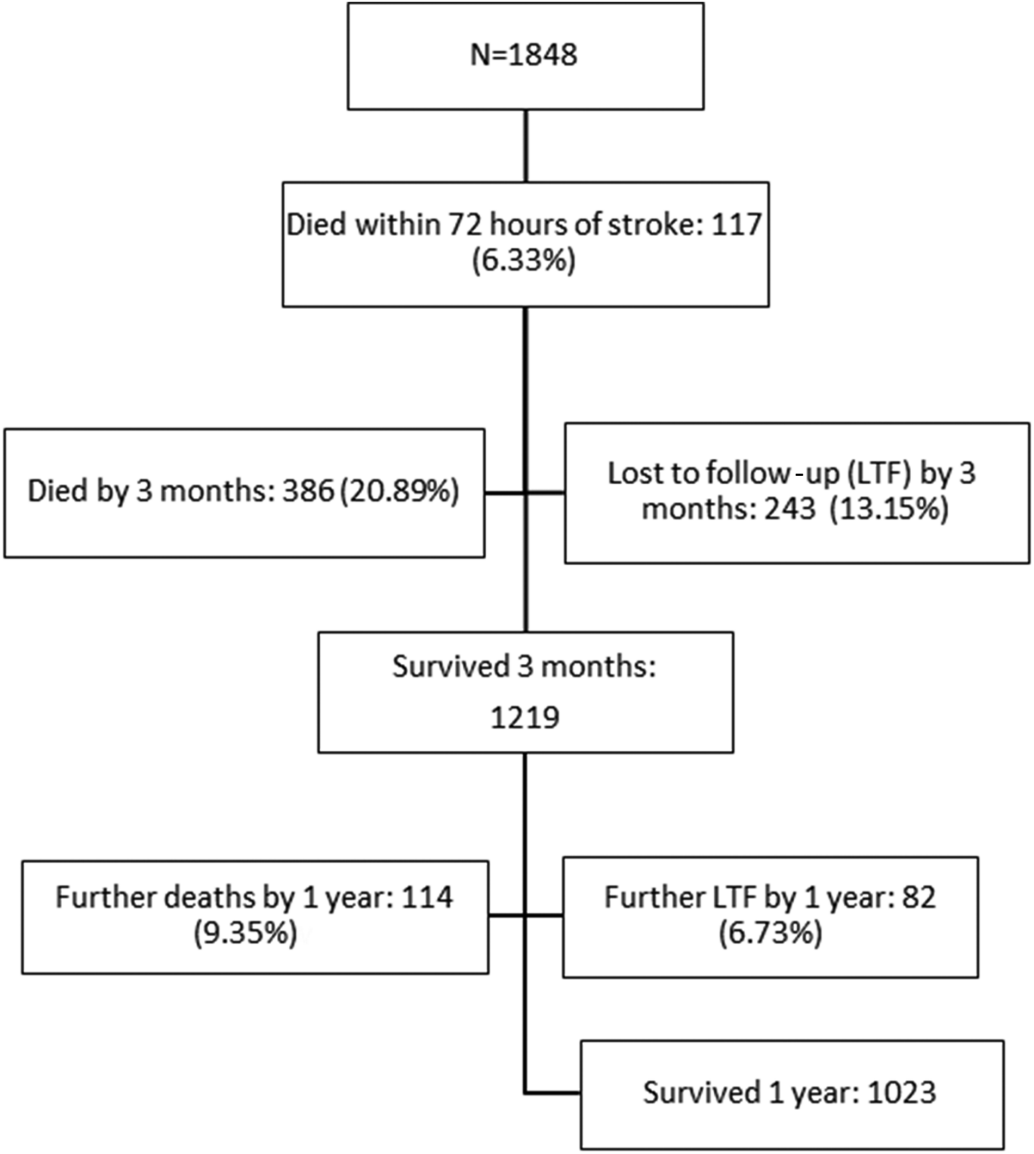
STROBE flowchart of registered patients with stroke in five European populations combined (N is the number of patients with cerebral infarction or intracerebral haemorrhage (ICH) interviewed).

The STROBE flow chart (figure 1) summarises the cohort follow-up including deaths within 72 h, 3 months and 1 year after stroke for the five populations combined. Details of these by population are available in the online supplementary table S1. Patients’ baseline characteristics, severity measures in the acute phase, and their PCS and MCS at 3 months and 1 year after stroke are given in table 1.

**Table 1:** Demography and severity measures at acute phase, and SF-12 components at 3 months and 1 year after stroke

	London		Kaunas		Warsaw		Dijon		Sesto Fiorentino		All	
N	425		792		118		368		145		1848	
Age, mean (SD)	69.6	(14.5)	70.8	(12.5)	70.4	(13.0)	74.4	(13.2)	75.7	(11.7)	71.7	(13.2)
	n	%	n	%	n	%	n	%	n	%	n	%
Female	236	55.5	341	43.1	58	49.2	180	48.9	75	51.7	890	48.2
Male	189	44.5	451	56.9	60	50.8	188	51.1	70	48.3	958	51.8
GCS (verbal component)												
Bad response	178	41.9	297	37.5	36	30.5	65	17.7	38	26.2	614	33.2
Oriented	244	57.4	483	61	81	68.6	302	82.1	102	70.3	121	65.6
Unknown	3	0.7	12	1.5	1	0.8	1	0.3	5	3.4	22	1.2
Able to walk without help												
No	238	56	563	71.1	60	50.8	175	47.6	91	62.8	1127	61
Yes	185	43.5	212	26.8	57	48.3	187	50.8	54	37.2	695	37.6
Unknown	2	0.5	17	2.1	1	0.8	6	1.6	0	0	26	1.4
Able to lift arms												
No	191	44.9	454	57.3	47	39.8	141	38.3	66	45.5	899	48.6
Yes	233	54.8	322	40.7	70	59.3	220	59.8	79	54.5	924	50
Unknown	1	0.2	16	2	1	0.8	7	1.9	0	0	25	1.4
BI												
Not independent (BI≤19)	76	17.9	120	15.2	31	26.3	77	20.9	33	22.8	337	18.2
Independent (BI=20)	347	81.7	666	84.1	66	55.9	254	69.0	110	75.9	1443	78.1
Unknown	2	0.5	6	0.8	21	17.8	37	10.1	2	1.4	68	3.7
Swallow test												
Fail	137	32.2	125	15.8	17	14.4	65	17.7	30	20.7	374	20.2
Pass	254	59.8	584	73.7	89	75.4	274	74.5	80	55.2	1281	69.3
Unknown	34	8	83	10.5	12	10.2	29	7.9	35	24.1	193	10.4
Incontinent/catheterised												
No	284	66.8	546	68.9	82	69.5	317	86.1	88	60.7	1317	71.3
Yes	102	24	150	18.9	15	12.7	37	10.1	47	32.4	351	19.0
Unknown	39	9.2	96	12.1	21	17.8	14	3.8	10	6.9	180	9.7
Completed SF-12 at 3 months, n	191		471		222		84		89		1057	
3 months PCS, mean (SD)	37.5	(11.1)	34.3	(9.6)	41.3	(10.7)	38.8	(11.9)	41.6	(10.4)	37.0	(10.8)
1 year PCS, mean (SD)	38.6	(11.0)	39.8	(10.7)	40.9	(11.0)	37.5	(13.7)	44.5	(10.9)	39.7	(11.5)
3 months MCS, mean (SD)	47.3	(12.1)	51.2	(11.5)	46.7	(10.7)	39.8	(9.4)	44.2	(11.3)	47.2	(12.0)
1 year MCS, mean (SD)	49.0	(11.2)	53.6	(9.1)	49.0	(10.5)	37.8	(7.3)	43.5	(10.5)	48.7	(11.1)

Differences across populations for all variables were significant at p value <0.001.

BI, Barthel Index; GCS, Glasgow Coma Scale; MCS, mental component summary; PCS, physical component summary; SF-12, Short-Form Health Survey.

The unadjusted ORs showed no differences between populations with exception of Dijon, which had a significantly lower risk of mortality within 3 months (see online supplementary table S2). Adjustment for stroke severity and age has altered the estimates considerably. The adjusted ORs of death within 3 months and 1 year of stroke are shown in table 2.

**Table 2:** Adjusted OR and 95% CI for death at 3 months and at 1 year after stroke in five European populations for all patients and excluding deaths within 72 h of stroke

	All registered patients				Excluding deaths within 72 h of stroke			
	OR	(95% CI)		p Value	OR	(95% CI)		p Value
Death at 3 months								
London	1.00				1.00			
Kaunas	2.34	1.32	4.14	0.004	2.27	1.28	4.02	0.005
Warsaw	3.81	1.31	11.06	0.014	3.63	1.26	10.50	0.017
Dijon	1.51	0.71	3.22	0.287	1.11	0.50	2.48	0.792
Sesto Fiorentino	0.84	0.36	1.98	0.689	0.84	0.35	1.97	0.681
Death at 1 year								
London	1.00				1.00			
Kaunas	1.93	1.20	3.10	0.007	1.90	1.18	3.05	0.008
Warsaw	1.91	0.81	4.47	0.139	1.86	0.79	4.35	0.154
Dijon	2.17	1.20	3.92	0.010	1.96	1.07	3.58	0.029
Sesto Fiorentino	0.54	0.26	1.13	0.102	0.54	0.26	1.13	0.102

Estimates of ORs of the five populations were derived from a multivariable logistic regression model adjusting for age and severity measures including the verbal components of Glasgow Coma Scale (speech orientation); arm power; ability to walk; swallowing; incontinent or being catheterised in the acute phase.

Kaunas and Warsaw had an increased risk of mortality at 3 months after stroke compared with London. Other populations showed no significant differences from London. Excluding early deaths (within 72 h after stroke) made no major difference. For 1 year after stroke, the adjusted ORs showed the risk in Kaunas remained higher compared with other centres. Dijon also has a significantly higher risk of death within 1 year after the stroke. When deaths within 72 h of stroke were excluded, the risk of death for Warsaw was slightly attenuated and remained insignificant; the risk for Dijon was attenuated and remained significantly higher than London.

### Variations in quality of life at 3 months and 1 year after stroke

The mean PCS and MCS of SF-12 varied widely across populations. PCS was lowest in Kaunas (mean 34.3, SD=9.6) and highest in Sesto Fiorentino (mean 41.6, SD=10.4). MCS was lowest in Dijon (mean 39.8, SD=9.4) and highest in Kaunas (mean 51.2, SD=11.5) at 3 months after stroke and similar differences were observed at 1 year (table 1).

**Figure 2:**
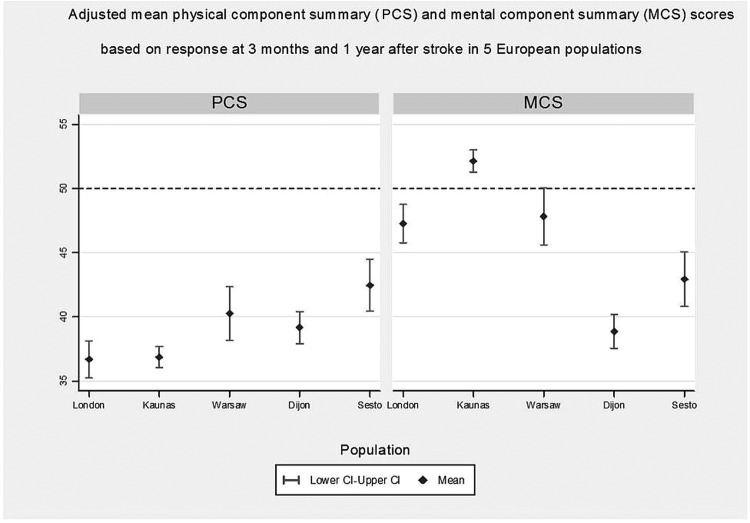
The left and right panels show adjusted means of the physical component summary (PCS) and mental component summary (MCS) scores, respectively, and their 95% CIs.

The unadjusted estimates of PCS and MCS differences between populations are given in online supplementary table S3. The size of differences for the five populations was altered after adjusting for severity and case mix but the order remained; Sesto Fiorentino, for example, had a significantly highest PCS score, a difference of 5.79 (p value <0.001) higher than London. For the MCS, Dijon had the lowest adjusted mean, with a difference that was 8.39 (p value <0.001) lower than that for London, while that for Kaunas was significantly higher than London, a difference of 4.88 (p values <0.001), table 3. Adjusted means are displayed in figure 2.

**Table 3:** Adjusted differences of the PCS and the MCS of SF-12, and other risk factors for the five populations

	Physical health composite score (PCS)				Mental health composite score (MCS)			
	Difference	95% CI		p Value	Difference	95% CI		p Value
London	(reference)							
Kaunas	0.19	−1.48	1.86	0.827	4.88	3.15	6.60	0.000
Warsaw	3.57	1.02	6.12	0.006	0.57	−2.10	3.23	0.677
Dijon	2.48	0.56	4.41	0.012	−8.39	−10.39	−6.40	0.000
Sesto Fiorentino	5.79	3.28	8.29	0.000	−4.33	−6.94	−1.71	0.001
Age	−0.25	−0.29	−0.20	0.000	−0.05	−0.10	0.00	0.037
GCS (verbal component score 5)	1.77	0.20	3.34	0.027	3.58	1.94	5.22	0.000
Able to walk without help	3.83	2.50	5.15	0.000	1.76	0.37	3.16	0.013
Able to lift arms horizontally	3.88	2.51	5.25	0.000	1.41	−0.03	2.85	0.055
Physically independent (BI=20)	3.61	−0.66	7.88	0.098	1.76	−2.71	6.23	0.440
Pass swallow test	0.42	−1.25	2.08	0.625	1.10	−0.64	2.85	0.216
Incontinent/catheterised	−4.27	−6.96	−1.58	0.002	−4.68	−7.36	−2.01	0.001

Estimates were obtained using a mixed effect regression model based on repeated measures at 3 months and 1 year after stroke. Adjustment was made for age and severity measures including the verbal components of GCS (speech orientation); arm power; ability to walk; swallowing; incontinent or being catheterised in the acute phase. Estimates were approximated to two decimal places, and p values were approximated to three decimal places, ‘0’ may represent a very small positive or negative value that has been approximated.

GCS <5: not fully oriented; GCS score 5: oriented speech.

BI, Barthel Index; GCS, Glasgow Coma Scale; MCS, mental component summary; PCS, physical component summary; SF-12, Short-Form Health Survey.

Older age, physical dependency, poor arm power, being unconscious, and being incontinent in the acute phase were strong determinants of poor PCS and MCS. The degree of association is, however, different for each outcome. For example, while a 1 year increase in age was associated with a reduction by 0.25 units of mean PCS (p value <0.001), the corresponding reduction in mean MCS was much smaller, 0.05 (p value=0.04).

The percentages of responses by levels of each dimension of the EQ-5D are presented for each of the populations in the online supplementary table S4A, B, for 3 months and 1 year after stroke, respectively. There were wide differences for each level of problems across populations in all five dimensions of the EQ-5D. For example, 49.7% of patients in London had no problems with mobility at 3 months after stroke; the corresponding figures were 41.8%, 49.8%, 58.3% and 59.6% in Kaunas, Warsaw, Dijon and Sesto Fiorentino, respectively. Similar differences were observed at 1 year after stroke and these were observed for all EQ-5D. No p values were considered to assess significance due to the large number of comparisons and the inflation of type I error. A few selected comparisons were made, which were significant at the 1% level.

**Figure 3:**
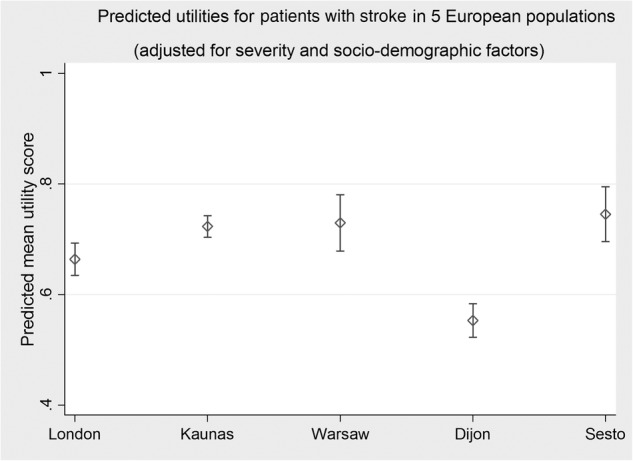
The figure shows the adjusted mean utilities and their 95% CIs.

Examination of the quality of life (QoL), as measured by utility scores, also showed differences between populations; these differences remained after adjustments were made. Sesto Fiorentino had significantly higher utility scores and Dijon had significantly lower scores compared with London, while no differences between London and Warsaw were observed. The adjusted mean utility scores are displayed in figure 3.

### Associations between reported HRQoL at 3 months and 1 year survival

Patients’ perception of HRQoL at 3 months was a strong predictor of survival within a year of stroke. The unadjusted ORs were significant for the two levels: some problems and extreme problems in all EQ-5D, with extreme problems being highly significant (p<0.001) (see online supplementary table S5). Adjustment attenuated these associations, but most remained significant (table 4). For example, reporting extreme problems with activity, pain, and anxiety and depression compared with reporting no problems have the following ORs for 1 year mortality: 6.38 (2.11 to 19.29), 3.33 (1.08 to 10.26) and 3.43 (1.30 to 9.03), for the three dimensions, respectively.

**Table 4:** Adjusted OR and 95% CIs for death by 1 year for three levels of response to EQ-5D at 3 months after stroke

	OR	(95% CI)		p Value
Activity				
No problem	1.0			
Some problems	3.35	1.29	8.68	0.013
Extreme problems	6.38	2.11	19.29	0.001
Pain				
No problem	1.0			
Some problems	1.12	0.44	2.86	0.807
Extreme problems	3.33	1.08	10.26	0.036
Anxiety and depression				
No problem	1.0			
Some problems	2.07	0.93	4.58	0.073
Extreme problems	3.43	1.30	9.03	0.013
Mobility				
No problem	1.0			
Some problems	2.75	1.26	5.98	0.011
Extreme problems	4.38	1.15	16.63	0.03
Self-care				
No problem	1.0			
Some problems	2.13	1.00	4.52	0.05
Extreme problems	1.35	0.46	3.96	0.58

For these three dimensions, the association of each with mortality was independent and inclusion of other dimensions in the model did not alter the magnitudes or the significance of these estimates. For mobility and self-care dimensions, the associations were, however, influenced by the inclusion of activity and pain dimensions in the model and in the presence of the latter, the former (mobility and self-care) became insignificant. For that reason we included mobility and self-care dimensions one at a time, in a model that adjusted for severity and case mix but does not include the other EQ-5D. Reporting some or extreme problems in mobility, was found to be significantly associated with death; while for self-care, the association was border line for reporting some problems, only. These estimates were obtained from final full models that take into account age, severity measures and population as previously reported. Data were confined to those who survived the 3 months and completed the SF-12. The odds of death within a year of stroke for patients who reported extreme problems, some problems and no problems, revealed a dose–response relationship for all dimensions except self-care.

## Discussion

The study demonstrated significant variations in survival and HRQoL at 3 months and 1 year after stroke across five European populations that could not be explained by stroke severity and sociodemographic factors. Earlier studies have reported differences in stroke outcomes, including incidence, survival, and HRQoL within and across geographical regions.^11^ ^29–32^ The differences have often been attributed to methodological problems such as confounding bias, different measures and methods of assessment, the lack of standardised procedures, and variations in care.^31^ ^33^ The variations observed in the current study seem to agree with these and remain unexplained, given the standardisation of training, methods of assessment of baseline information, including case mix and sociodemographic and protocoled assessments at 3 and 12 months.

About half of stroke survivors reported symptoms of anxiety and depression, and limitations in activity and mobility, while over 70% reported experiencing pain or discomfort; highlighting the impact of stroke on different aspects of QoL. Wide variations across populations were observed in every dimension covered by the EQ-5D. Reporting poor HRQoL was significantly associated with increased chances of death within 1 year of stroke, enhancing evidence on the value of self-reported health for prognosis and for clinical decision-making.^34^

The variations in survival were partially, but not fully, attributed to stroke severity. In general, 1 year survivors were more likely to be younger, have better arm power, were able to walk without help from another person, remained conscious and were not incontinent or catheterised during the acute phase. Most of these factors have been previously reported as predictors of survival or independent survival after stroke.^35^ The consistently lower survival in Kaunas (Lithuania) might be attributed to health system-related problems that have been reported, including health workers’ dissatisfaction and bureaucracy that could impact negatively on quality of care and therefore, on patients’ outcomes.^36^ One reason for differences in PROMs might be that self-reported health varies by culture and ethnicity, although the evidence that supports this is inconsistent and often contradictory.^37–39^ This may be worthy of further exploration within the European setting if further international comparisons are to be fully understood.

Overall the mean physical function observed is well below that of the general population and mental well-being was reduced in agreement with findings in young adults with ischaemic stroke.^40^ There are, however, variations between populations. In Kaunas, the mental domain score was fairly comparable to that of the general population estimates, which was rather unexpected. The higher score there may however be attributed to the better psychological well-being among those who survived 3 months, and a greater mortality for patients with stroke particularly the most disabled and possibly the more mentally or emotionally affected.

The higher adjusted mortality rates in Dijon, within a year of stroke, despite the highly rated healthcare system and the generally good case mix in the acute phase, may be attributed to the higher proportion of participants lost at follow-up in Dijon, and the possible speculation that loss at follow-up may be more common among healthier patients. The association between mortality and poor-rated health found in this study are consistent with findings in stroke and other health conditions’ research.^3^

### Strengths

A lack of standardisation of procedures across regions has consistently been described as a hindrance to comparisons between populations. The major strength of this study is the use of standard measures and methods for data collection across all populations that would control for many common biases of trials and hospital-based studies.^41^ To the best of our knowledge, no study has been conducted following ours that has covered as many aspects of health, mortality, disability and QoL as this one has, suggesting that our data may be a useful source of information on differences between European populations.

Mapping SF-12 into EQ-5D was particularly useful in providing insights into differences in patient-assessed health in addition to those provided by more clinical measures, and by the PCS and MCS. The magnitude of the utility scores on the overall was comparable with those derived from other similar studies and provides additional information that may be used to undertake economic evaluations in the future.^42^ The study complies with the STROBE statement.

### Limitations

Limitations include the relatively short follow-up period limiting the inference on survival and the associations of perception of health and survival up to 1 year after stroke. The use of the generic scale SF-12, rather than a stroke-specific scale, may not have captured all aspects of social functioning that are meaningful to patients with stroke.^43^ There is, however, strong support for the use of SF-12 in assessing the health of patients with stroke.^44^

The observational nature of the study made it open to possible recognised biases due to unmeasured confounders, such as behavioural and social factors, that may affect the life course but are unmeasured or unknown. The selection of the study populations was based on previous experience in running stroke registers and these may not necessarily be representative of their countries as a whole. The EQ-5D-based estimates used in this study were generated from the SF-12 responses, not from responses to the EQ-5D itself. These are, therefore, subject to limitations that may result from how the data are mapped.^23^

### Conclusions

These data indicate that mortality and HRQoL 3 months and 1 year after stroke varied significantly across five European populations. Despite standardised methods of measurement, there remain unexplained differences in mortality, and the physical and mental well-being of stroke survivors after controlling for stroke severity or case mix. Differences in cultural factors, health systems and resource availability might be partially responsible for these differences. Poor HRQoL at 3 months was significantly associated with increased risk of death within 1 year of stroke, supporting the value of PROMs for both prognosis, and clinical decision-making. The findings may be generalisable to other long-term conditions.
